# *catena*-Poly[hemi[bis­(4′-phenyl-2,2′:6′,2′′-terpyridine-κ^3^ *N*)copper(II)] [cuprate(I)-di-μ_2_-thio­cyanato-κ^2^ *N*:*S*;κ^2^ *S*:*N*]]

**DOI:** 10.1107/S1600536809023009

**Published:** 2009-06-20

**Authors:** Wen-Juan Shi

**Affiliations:** a Jiangxi Key Laboratory of Surface Engineering, Jiangxi Science and Technology Normal University, Jiangxi 330013, People’s Republic of China

## Abstract

Reaction of 4′-phenyl-2,2′:6′,2′′-terpyridine (phtpy), copper acetate hydrate and ammonium thio­cyanate under solvothermal conditions led to the formation of the title compound, {[Cu(C_21_H_15_N_3_)_2_][Cu_2_(NCS)_4_]}_*n*_. The structure is composed of discrete [Cu(phtpy)_2_]^2+^ cations and polymeric anionic {[Cu(SCN)_2_]^−^} chains propagating along [010]. The central Cu^2+^ ion in the cation is coordinated by two tridentate chelating phtpy ligands in a distorted octa­hedral geometry. In each of the two crystallographically independent centrosymmetric anions, the Cu^I^ atoms are bridged in a 1,3-μ_2_-bridging mode by two S and two N atoms, resulting in a distorted tetrahedral CuN_2_S_2_ coordination. The [Cu(phtpy)_2_]^2+^ cations are fixed between these polymers by inter­molecular C—H⋯S hydrogen bonds.

## Related literature

For related 2,2′:6′,2′′-terpyridine derivatives and their complexes, see: Heller & Schubert (2003 ▶); Hofmeier & Schubert (2004 ▶); Shi *et al.* (2007 ▶). For the isostructural 4′-(3-pyridyl)-2,2′:6′,2′′-terpyridine (3-pytpy) analogue, see: Shi (2009 ▶).
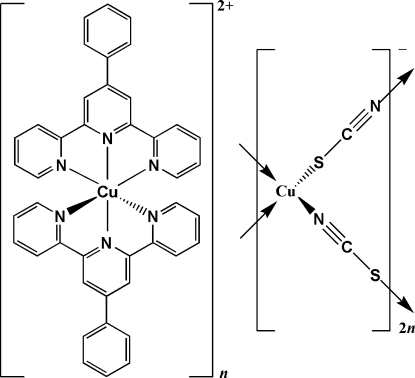


## Experimental

### 

#### Crystal data

[Cu(C_21_H_15_N_3_)_2_][Cu_2_(NCS)_4_]
*M* *_r_* = 1041.66
Triclinic, 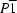

*a* = 10.1803 (6) Å
*b* = 10.1829 (6) Å
*c* = 21.3203 (12) Å
α = 83.571 (1)°
β = 89.566 (1)°
γ = 81.676 (1)°
*V* = 2173.0 (2) Å^3^
*Z* = 2
Mo *K*α radiation
μ = 1.70 mm^−1^
*T* = 295 K
0.15 × 0.14 × 0.12 mm

#### Data collection

Bruker SMART APEX area-detector diffractometer
Absorption correction: multi-scan (*SADABS*; Sheldrick, 1996 ▶) *T*_min_ = 0.785, *T*_max_ = 0.823
17217 measured reflections
8453 independent reflections
5988 reflections with *I* > 2σ(*I*)
*R*_int_ = 0.031

#### Refinement

*R*[*F* ^2^ > 2σ(*F* ^2^)] = 0.055
*wR*(*F* ^2^) = 0.141
*S* = 1.03
8453 reflections
568 parameters
54 restraints
H-atom parameters constrained
Δρ_max_ = 1.53 e Å^−3^
Δρ_min_ = −0.89 e Å^−3^

### 

Data collection: *SMART* (Bruker, 2002 ▶); cell refinement: *SAINT* (Bruker, 2002 ▶); data reduction: *SAINT*; program(s) used to solve structure: *SHELXS97* (Sheldrick, 2008 ▶); program(s) used to refine structure: *SHELXL97* (Sheldrick, 2008 ▶); molecular graphics: *SHELXTL* (Sheldrick, 2008 ▶); software used to prepare material for publication: *SHELXTL*.

## Supplementary Material

Crystal structure: contains datablocks I, global. DOI: 10.1107/S1600536809023009/at2820sup1.cif

Structure factors: contains datablocks I. DOI: 10.1107/S1600536809023009/at2820Isup2.hkl

Additional supplementary materials:  crystallographic information; 3D view; checkCIF report

## Figures and Tables

**Table 1:** Hydrogen-bond geometry (Å, °)

*D*—H⋯*A*	*D*—H	H⋯*A*	*D*⋯*A*	*D*—H⋯*A*
C28—H28⋯S3^i^	0.93	2.82	3.697 (5)	158
C36—H36⋯S1	0.93	2.81	3.657 (5)	152

Symmetry code: (i) 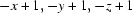.

**Fig. 1.**
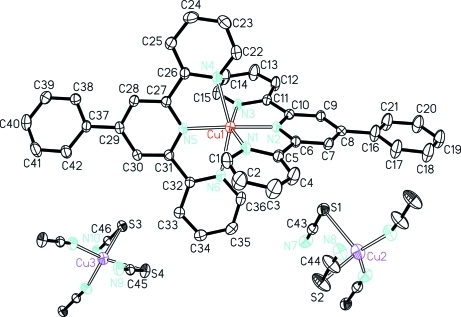
The title compound with displacement ellipsoids drawn at the 30% probability level. The H atoms are omitted for clarity.

**Fig. 2.**
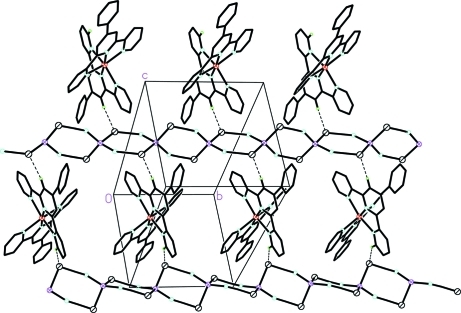
A packing diagram of the title compound, showing the intermolecular C–H···S hydrogen bonds as dashed lines. The H atoms not involved in hydrogen bonds have been omitted for clarity.

## supplementary crystallographic information

### Comment

2,2':6',2''-Terpyridine and its derivatives have attracted considerable interest as ligands in metal complexes because of their versatility as building blocks for supramolecular assembles and polymers (Heller & Schubert, 2003; Hofmeier & Schubert, 2004). As a continuing effort of our research on complexes of terpyridine derivatives (Shi *et al.*, 2007), we here report the title compound by using 4'-phenyl-2,2':6',2''-terpyridine (phtpy) as ligand, containing discrete cations [Cu(phtpy)_2_]^2+^ and one-dimensional polymeric anionic chains [Cu(SCN)_2_]_n_^n-^.

As shown in Fig. 1, the molecular structure of the title compound consists of three independent fragments. The central Cu^2+^ ion in the cation is coordinated by two tridentate chelating phtpy ligands to form a distorted octahedral geometry. The phtpy ligands are approximately orthogonal to one another, with a dihedral angle of 76.4 (2) ° between planes through the three six-membered rings of the two ligands. The pendent and central pyridine in one phtpy are almost coplanar with a dihedral angle of 1.4 (1) °, but in the other phtpy, a corresponding serious tilt of 35.3 (2) ° is formed. There exists two independent crystallographically centrosymmetric [Cu(SCN)_2_]^-^ anions, in which each Cu^+^ centre is coordinated by two S atoms and two N atoms from four isothiocyanate ligands with a distorted tetrahedron geometry. Each isothiocyanate anion acts as a 1,3-µ_2_ bridging ligand to bridge two Cu^+^ ions, resulting in the formation of polymeric [Cu(SCN)_2_]_n_^n-^ anionic chains. All the SCN^-^ groups are almost linear with the S–C–N bond angles in the range 176.3 (7) °–179.1 (4) °.

Two –CH groups in the [Cu(phtpy)_2_]^2+^ cation interact with two S atoms form two polymeric [Cu(SCN)_2_]_n_^n-^ anionic chains through C–H···S hydrogen bonds [C···S = 3.697 (5) Å and 3.657 (5) Å, respectively] to form a two-dimensional supramolecular array (Fig.ure 2).

### Experimental

A mixture of copper acetate hydrate (39.9 mg, 0.20 mmol), phtpy (30.9 mg, 0.10 mmol) and ammonium thiocyanate (15.2 mg, 0.20 mmol) in ethanol (12 ml) was sealed in a 15 ml Teflon-lined reactor, heated to 423 K for 72 h, and then cooled to room temperature at a rate of 6 K/h to give black crystals of the title compound [yield: 12 mg (22%)].

### Refinement

The carbon-bound H atoms were placed at calculated positions (C—H = 0.93 Å) and refined as riding, with *U*(H) = 1.2*U*_eq_(C).

### Crystal data

**Table:**

[Cu(C_21_H_15_N_3_)_2_][Cu_2_(NCS)_4_]	*Z* = 2
*M**_r_* = 1041.66	*F*(000) = 1054
Triclinic, *P*1	*D*_x_ = 1.592 Mg m^−^^3^
Hall symbol: -P 1	Mo *K*α radiation, λ = 0.71073 Å
*a* = 10.1803 (6) Å	Cell parameters from 3113 reflections
*b* = 10.1829 (6) Å	θ = 2.6–22.6°
*c* = 21.3203 (12) Å	µ = 1.70 mm^−^^1^
α = 83.571 (1)°	*T* = 295 K
β = 89.566 (1)°	Block, black
γ = 81.676 (1)°	0.15 × 0.14 × 0.12 mm
*V* = 2173.0 (2) Å^3^	

### Data collection

**Table:**

Bruker SMART APEX area-detector diffractometer	8453 independent reflections
Radiation source: fine-focus sealed tube	5988 reflections with *I* > 2σ(*I*)
graphite	*R*_int_ = 0.031
φ and ω scans	θ_max_ = 26.0°, θ_min_ = 2.0°
Absorption correction: multi-scan (*SADABS*; Sheldrick, 1996)	*h* = −12→12
*T*_min_ = 0.785, *T*_max_ = 0.823	*k* = −12→12
17217 measured reflections	*l* = −26→26

### Refinement

**Table:**

Refinement on *F*^2^	Primary atom site location: structure-invariant direct methods
Least-squares matrix: full	Secondary atom site location: difference Fourier map
*R*[*F*^2^ > 2σ(*F*^2^)] = 0.055	Hydrogen site location: inferred from neighbouring sites
*wR*(*F*^2^) = 0.141	H-atom parameters constrained
*S* = 1.03	*w* = 1/[σ^2^(*F*_o_^2^) + (0.0609*P*)^2^ + 1.919*P*] where *P* = (*F*_o_^2^ + 2*F*_c_^2^)/3
8453 reflections	(Δ/σ)_max_ = 0.001
568 parameters	Δρ_max_ = 1.53 e Å^−^^3^
54 restraints	Δρ_min_ = −0.89 e Å^−^^3^

### Special details

**Table:**

Geometry. All e.s.d.'s (except the e.s.d. in the dihedral angle between two l.s. planes) are estimated using the full covariance matrix. The cell e.s.d.'s are taken into account individually in the estimation of e.s.d.'s in distances, angles and torsion angles; correlations between e.s.d.'s in cell parameters are only used when they are defined by crystal symmetry. An approximate (isotropic) treatment of cell e.s.d.'s is used for estimating e.s.d.'s involving l.s. planes.
Refinement. Refinement of *F*^2^ against ALL reflections. The weighted *R*-factor *wR* and goodness of fit *S* are based on *F*^2^, conventional *R*-factors *R* are based on *F*, with *F* set to zero for negative *F*^2^. The threshold expression of *F*^2^ > σ(*F*^2^) is used only for calculating *R*-factors(gt) *etc*. and is not relevant to the choice of reflections for refinement. *R*-factors based on *F*^2^ are statistically about twice as large as those based on *F*, and *R*- factors based on ALL data will be even larger.

### Fractional atomic coordinates and isotropic or equivalent isotropic displacement parameters (Å^2^)

**Table:**

	*x*	*y*	*z*	*U*_iso_*/*U*_eq_	
Cu1	0.49651 (5)	0.79721 (6)	0.75522 (2)	0.04334 (17)	
Cu2	−0.00482 (7)	0.74884 (6)	1.01693 (3)	0.0650 (2)	
Cu3	0.02122 (6)	0.24319 (6)	0.52085 (3)	0.05204 (18)	
S1	0.18721 (14)	0.64253 (13)	0.95859 (6)	0.0549 (3)	
S2	−0.14546 (18)	0.86946 (15)	0.93153 (9)	0.0860 (6)	
S3	0.19621 (12)	0.07262 (12)	0.56458 (6)	0.0464 (3)	
S4	−0.04694 (14)	0.35973 (13)	0.60866 (6)	0.0550 (3)	
N1	0.3676 (4)	0.9799 (4)	0.77294 (17)	0.0492 (9)	
N2	0.5013 (3)	0.7885 (4)	0.84804 (16)	0.0386 (8)	
N3	0.6371 (4)	0.6148 (4)	0.78198 (16)	0.0416 (9)	
N4	0.6732 (4)	0.8846 (4)	0.72222 (17)	0.0467 (9)	
N5	0.4877 (3)	0.7923 (3)	0.66328 (15)	0.0374 (8)	
N6	0.3130 (4)	0.7109 (4)	0.74383 (17)	0.0488 (10)	
N7	0.0858 (5)	0.4059 (4)	0.9483 (2)	0.0636 (12)	
N8	−0.0544 (5)	1.1122 (5)	0.9388 (2)	0.0630 (12)	
N9	−0.0682 (4)	0.6194 (4)	0.54712 (18)	0.0510 (10)	
N10	0.1066 (4)	−0.1400 (4)	0.51346 (19)	0.0504 (10)	
C1	0.2992 (6)	1.0712 (6)	0.7315 (2)	0.0692 (16)	
H1	0.3185	1.0694	0.6888	0.083*	
C2	0.2026 (7)	1.1665 (7)	0.7484 (3)	0.0863 (18)	
H2	0.1576	1.2291	0.7179	0.104*	
C3	0.1727 (7)	1.1693 (7)	0.8105 (3)	0.0926 (19)	
H3	0.1073	1.2342	0.8233	0.111*	
C4	0.2411 (6)	1.0740 (6)	0.8546 (3)	0.0749 (18)	
H4	0.2208	1.0729	0.8973	0.090*	
C5	0.3388 (5)	0.9815 (5)	0.8345 (2)	0.0450 (11)	
C6	0.4210 (4)	0.8776 (4)	0.87713 (19)	0.0378 (10)	
C7	0.4160 (4)	0.8682 (4)	0.94226 (19)	0.0401 (10)	
H7	0.3594	0.9312	0.9617	0.048*	
C8	0.4947 (4)	0.7653 (4)	0.97905 (19)	0.0379 (10)	
C9	0.5786 (4)	0.6746 (4)	0.94723 (19)	0.0390 (10)	
H9	0.6334	0.6047	0.9700	0.047*	
C10	0.5807 (4)	0.6884 (4)	0.8820 (2)	0.0384 (10)	
C11	0.6651 (4)	0.5962 (5)	0.84408 (19)	0.0404 (10)	
C12	0.7644 (5)	0.5007 (6)	0.8694 (2)	0.0631 (15)	
H12	0.7828	0.4908	0.9125	0.076*	
C13	0.8364 (6)	0.4198 (6)	0.8306 (3)	0.0768 (19)	
H13	0.9038	0.3536	0.8470	0.092*	
C14	0.8086 (6)	0.4371 (6)	0.7673 (2)	0.0657 (16)	
H14	0.8561	0.3826	0.7402	0.079*	
C15	0.7106 (5)	0.5350 (5)	0.7447 (2)	0.0535 (13)	
H15	0.6932	0.5477	0.7015	0.064*	
C16	0.4897 (5)	0.7524 (5)	1.0481 (2)	0.0674 (10)	
C17	0.4049 (6)	0.8408 (6)	1.0785 (2)	0.0786 (11)	
H17	0.3529	0.9114	1.0549	0.094*	
C18	0.3944 (6)	0.8278 (6)	1.1434 (2)	0.0832 (11)	
H18	0.3313	0.8861	1.1622	0.100*	
C19	0.4748 (6)	0.7313 (5)	1.1807 (2)	0.0796 (11)	
H19	0.4723	0.7269	1.2245	0.095*	
C20	0.5589 (6)	0.6414 (6)	1.1509 (2)	0.0803 (11)	
H20	0.6134	0.5730	1.1747	0.096*	
C21	0.5640 (6)	0.6510 (6)	1.08594 (19)	0.0762 (10)	
H21	0.6198	0.5864	1.0671	0.091*	
C22	0.7639 (6)	0.9315 (6)	0.7553 (2)	0.0603 (14)	
H22	0.7551	0.9281	0.7989	0.072*	
C23	0.8678 (6)	0.9834 (6)	0.7278 (3)	0.0698 (16)	
H23	0.9282	1.0165	0.7522	0.084*	
C24	0.8837 (6)	0.9871 (6)	0.6640 (3)	0.0709 (17)	
H24	0.9538	1.0242	0.6444	0.085*	
C25	0.7956 (5)	0.9358 (6)	0.6293 (2)	0.0581 (14)	
H25	0.8060	0.9352	0.5859	0.070*	
C26	0.6905 (4)	0.8847 (4)	0.6596 (2)	0.0413 (10)	
C27	0.5899 (4)	0.8247 (4)	0.62715 (19)	0.0378 (10)	
C28	0.5990 (5)	0.7969 (5)	0.5653 (2)	0.0429 (11)	
H28	0.6706	0.8186	0.5410	0.052*	
C29	0.5018 (4)	0.7367 (4)	0.53914 (19)	0.0405 (10)	
C30	0.3948 (4)	0.7099 (4)	0.57630 (19)	0.0374 (10)	
H30	0.3263	0.6729	0.5596	0.045*	
C31	0.3894 (4)	0.7382 (4)	0.63832 (19)	0.0374 (10)	
C32	0.2836 (4)	0.7071 (4)	0.68283 (19)	0.0394 (10)	
C33	0.1616 (5)	0.6776 (5)	0.6644 (2)	0.0490 (12)	
H33	0.1421	0.6768	0.6219	0.059*	
C34	0.0712 (5)	0.6499 (6)	0.7093 (3)	0.0665 (16)	
H34	−0.0103	0.6284	0.6978	0.080*	
C35	0.1010 (6)	0.6538 (6)	0.7718 (3)	0.0711 (17)	
H35	0.0399	0.6359	0.8030	0.085*	
C36	0.2221 (5)	0.6846 (6)	0.7870 (2)	0.0644 (15)	
H36	0.2420	0.6874	0.8292	0.077*	
C37	0.5151 (4)	0.6986 (5)	0.47397 (19)	0.0410 (10)	
C38	0.6401 (5)	0.6557 (4)	0.4509 (2)	0.0444 (11)	
H38	0.7146	0.6509	0.4765	0.053*	
C39	0.6547 (5)	0.6202 (5)	0.3905 (2)	0.0517 (12)	
H39	0.7389	0.5924	0.3755	0.062*	
C40	0.5463 (6)	0.6258 (6)	0.3528 (2)	0.0630 (15)	
H40	0.5567	0.6005	0.3123	0.076*	
C41	0.4217 (6)	0.6686 (6)	0.3744 (2)	0.0692 (16)	
H41	0.3480	0.6729	0.3483	0.083*	
C42	0.4056 (5)	0.7053 (5)	0.4349 (2)	0.0561 (13)	
H42	0.3211	0.7346	0.4493	0.067*	
C43	0.1256 (5)	0.5044 (5)	0.9518 (2)	0.0467 (11)	
C44	−0.0890 (7)	1.0120 (6)	0.9343 (3)	0.0790 (11)	
C45	−0.0607 (4)	0.5133 (5)	0.5729 (2)	0.0400 (9)	
C46	0.1439 (4)	−0.0529 (5)	0.5350 (2)	0.0397 (10)	

### Atomic displacement parameters (Å^2^)

**Table:**

	*U*^11^	*U*^22^	*U*^33^	*U*^12^	*U*^13^	*U*^23^
Cu1	0.0446 (3)	0.0535 (4)	0.0328 (3)	−0.0080 (3)	0.0034 (2)	−0.0079 (2)
Cu2	0.0847 (5)	0.0437 (4)	0.0693 (5)	−0.0180 (3)	−0.0072 (4)	−0.0058 (3)
Cu3	0.0624 (4)	0.0423 (3)	0.0525 (4)	−0.0099 (3)	−0.0023 (3)	−0.0068 (3)
S1	0.0606 (8)	0.0564 (8)	0.0512 (7)	−0.0212 (6)	0.0085 (6)	−0.0042 (6)
S2	0.1016 (13)	0.0481 (8)	0.1102 (13)	−0.0130 (8)	−0.0552 (10)	−0.0106 (8)
S3	0.0529 (7)	0.0451 (6)	0.0422 (6)	−0.0108 (5)	−0.0074 (5)	−0.0036 (5)
S4	0.0766 (9)	0.0474 (7)	0.0393 (6)	−0.0059 (6)	0.0072 (6)	−0.0018 (5)
N1	0.052 (2)	0.060 (2)	0.033 (2)	−0.0001 (15)	−0.0021 (18)	−0.0061 (18)
N2	0.036 (2)	0.049 (2)	0.0306 (19)	−0.0035 (17)	0.0009 (15)	−0.0069 (16)
N3	0.044 (2)	0.049 (2)	0.0314 (19)	−0.0029 (18)	0.0034 (16)	−0.0082 (17)
N4	0.056 (2)	0.055 (2)	0.032 (2)	−0.010 (2)	0.0024 (18)	−0.0116 (18)
N5	0.041 (2)	0.044 (2)	0.0280 (18)	−0.0059 (17)	−0.0011 (16)	−0.0059 (15)
N6	0.047 (2)	0.069 (3)	0.030 (2)	−0.008 (2)	−0.0007 (17)	−0.0061 (18)
N7	0.068 (3)	0.050 (3)	0.075 (3)	−0.017 (2)	0.016 (2)	−0.011 (2)
N8	0.078 (3)	0.056 (3)	0.055 (3)	−0.010 (2)	−0.013 (2)	−0.007 (2)
N9	0.064 (3)	0.045 (2)	0.044 (2)	−0.006 (2)	−0.002 (2)	−0.0060 (19)
N10	0.050 (2)	0.045 (2)	0.055 (3)	−0.0014 (19)	−0.011 (2)	−0.0045 (19)
C1	0.078 (4)	0.092 (4)	0.035 (3)	−0.016 (4)	−0.009 (3)	0.008 (3)
C2	0.086 (4)	0.097 (4)	0.058 (3)	0.026 (3)	−0.004 (3)	0.019 (3)
C3	0.090 (4)	0.102 (4)	0.064 (3)	0.041 (3)	0.004 (3)	0.015 (3)
C4	0.078 (4)	0.088 (4)	0.043 (3)	0.033 (3)	0.009 (3)	0.003 (3)
C5	0.044 (3)	0.055 (3)	0.034 (2)	0.000 (2)	0.003 (2)	−0.005 (2)
C6	0.038 (2)	0.044 (3)	0.029 (2)	0.001 (2)	−0.0004 (18)	−0.0059 (19)
C7	0.041 (3)	0.044 (3)	0.033 (2)	0.003 (2)	0.0061 (19)	−0.0080 (19)
C8	0.034 (2)	0.047 (3)	0.033 (2)	−0.010 (2)	−0.0011 (18)	−0.0068 (19)
C9	0.040 (2)	0.046 (3)	0.029 (2)	0.000 (2)	−0.0009 (18)	−0.0055 (19)
C10	0.035 (2)	0.046 (3)	0.034 (2)	−0.002 (2)	0.0022 (19)	−0.0089 (19)
C11	0.040 (2)	0.050 (3)	0.031 (2)	−0.005 (2)	0.0024 (19)	−0.006 (2)
C12	0.064 (3)	0.087 (4)	0.029 (3)	0.021 (3)	−0.001 (2)	−0.007 (3)
C13	0.079 (4)	0.091 (4)	0.046 (3)	0.036 (3)	0.004 (3)	−0.007 (3)
C14	0.082 (4)	0.068 (4)	0.044 (3)	0.011 (3)	0.017 (3)	−0.020 (3)
C15	0.072 (4)	0.061 (3)	0.029 (2)	−0.008 (3)	0.008 (2)	−0.013 (2)
C16	0.080 (2)	0.080 (2)	0.0313 (15)	0.0226 (18)	0.0027 (15)	−0.0060 (15)
C17	0.094 (2)	0.090 (2)	0.0370 (16)	0.0330 (18)	0.0063 (16)	−0.0037 (16)
C18	0.101 (2)	0.095 (2)	0.0386 (16)	0.0338 (19)	0.0078 (17)	−0.0060 (16)
C19	0.096 (2)	0.094 (2)	0.0361 (16)	0.0263 (19)	0.0032 (16)	−0.0038 (16)
C20	0.093 (2)	0.095 (2)	0.0380 (16)	0.0294 (19)	0.0019 (17)	−0.0005 (16)
C21	0.088 (2)	0.090 (2)	0.0373 (16)	0.0297 (18)	0.0028 (16)	−0.0028 (16)
C22	0.070 (4)	0.076 (4)	0.039 (3)	−0.015 (3)	0.000 (3)	−0.019 (3)
C23	0.060 (4)	0.093 (4)	0.064 (4)	−0.023 (3)	−0.008 (3)	−0.028 (3)
C24	0.060 (4)	0.088 (4)	0.076 (4)	−0.042 (3)	0.017 (3)	−0.020 (3)
C25	0.062 (3)	0.078 (4)	0.043 (3)	−0.031 (3)	0.011 (2)	−0.014 (3)
C26	0.048 (3)	0.047 (3)	0.030 (2)	−0.011 (2)	0.002 (2)	−0.0064 (19)
C27	0.041 (2)	0.042 (2)	0.031 (2)	−0.009 (2)	0.0021 (19)	−0.0033 (18)
C28	0.048 (3)	0.053 (3)	0.030 (2)	−0.015 (2)	0.004 (2)	−0.004 (2)
C29	0.048 (3)	0.044 (3)	0.029 (2)	−0.005 (2)	−0.0034 (19)	−0.0037 (19)
C30	0.039 (2)	0.044 (2)	0.030 (2)	−0.005 (2)	−0.0042 (18)	−0.0023 (18)
C31	0.038 (2)	0.039 (2)	0.032 (2)	−0.0018 (19)	−0.0011 (19)	0.0013 (18)
C32	0.038 (2)	0.047 (3)	0.033 (2)	−0.004 (2)	0.0006 (19)	−0.0060 (19)
C33	0.045 (3)	0.065 (3)	0.039 (3)	−0.014 (2)	0.000 (2)	−0.004 (2)
C34	0.049 (3)	0.101 (5)	0.055 (3)	−0.027 (3)	0.007 (3)	−0.013 (3)
C35	0.057 (4)	0.108 (5)	0.050 (3)	−0.024 (3)	0.016 (3)	0.001 (3)
C36	0.064 (4)	0.097 (4)	0.031 (3)	−0.012 (3)	0.007 (2)	−0.004 (3)
C37	0.048 (3)	0.048 (3)	0.027 (2)	−0.009 (2)	0.0019 (19)	−0.0034 (19)
C38	0.047 (3)	0.049 (3)	0.039 (2)	−0.010 (2)	0.001 (2)	−0.007 (2)
C39	0.057 (3)	0.054 (3)	0.045 (3)	−0.005 (2)	0.011 (2)	−0.011 (2)
C40	0.087 (4)	0.066 (4)	0.034 (3)	−0.002 (3)	0.001 (3)	−0.011 (2)
C41	0.072 (4)	0.093 (4)	0.043 (3)	0.001 (3)	−0.017 (3)	−0.019 (3)
C42	0.051 (3)	0.077 (4)	0.039 (3)	0.000 (3)	−0.005 (2)	−0.015 (2)
C43	0.048 (3)	0.049 (3)	0.041 (3)	−0.004 (2)	0.007 (2)	−0.001 (2)
C44	0.095 (2)	0.0446 (18)	0.098 (2)	−0.0090 (18)	−0.0519 (19)	−0.0091 (18)
C45	0.041 (2)	0.0463 (17)	0.032 (2)	−0.004 (2)	−0.0011 (19)	−0.0062 (16)
C46	0.038 (2)	0.044 (3)	0.034 (2)	0.001 (2)	−0.0030 (19)	0.002 (2)

### Geometric parameters (Å, °)

**Table:**

Cu1—N5	1.970 (3)	C11—C12	1.366 (6)
Cu1—N2	1.972 (3)	C12—C13	1.368 (7)
Cu1—N1	2.184 (4)	C12—H12	0.9300
Cu1—N4	2.197 (4)	C13—C14	1.368 (7)
Cu1—N3	2.198 (4)	C13—H13	0.9300
Cu1—N6	2.203 (4)	C14—C15	1.354 (7)
Cu2—N8^i^	1.953 (5)	C14—H14	0.9300
Cu2—N7^ii^	1.954 (4)	C15—H15	0.9300
Cu2—S2	2.4297 (17)	C16—C17	1.369 (4)
Cu2—S1	2.4886 (16)	C16—C21	1.373 (4)
Cu3—N10^iii^	1.979 (4)	C17—C18	1.378 (4)
Cu3—N9^iv^	2.004 (4)	C17—H17	0.9300
Cu3—S4	2.3738 (14)	C18—C19	1.368 (4)
Cu3—S3	2.4137 (13)	C18—H18	0.9300
S1—C43	1.642 (5)	C19—C20	1.367 (4)
S2—C44	1.643 (6)	C19—H19	0.9300
S3—C46	1.643 (5)	C20—C21	1.378 (4)
S4—C45	1.648 (5)	C20—H20	0.9300
N1—C1	1.330 (6)	C21—H21	0.9300
N1—C5	1.345 (5)	C22—C23	1.352 (7)
N2—C6	1.334 (5)	C22—H22	0.9300
N2—C10	1.350 (5)	C23—C24	1.366 (8)
N3—C11	1.343 (5)	C23—H23	0.9300
N3—C15	1.346 (6)	C24—C25	1.363 (7)
N4—C22	1.338 (6)	C24—H24	0.9300
N4—C26	1.345 (5)	C25—C26	1.382 (6)
N5—C27	1.347 (5)	C25—H25	0.9300
N5—C31	1.349 (5)	C26—C27	1.480 (6)
N6—C36	1.333 (6)	C27—C28	1.379 (6)
N6—C32	1.343 (5)	C28—C29	1.388 (6)
N7—C43	1.145 (6)	C28—H28	0.9300
N7—Cu2^ii^	1.954 (4)	C29—C30	1.384 (6)
N8—C44	1.141 (6)	C29—C37	1.484 (6)
N8—Cu2^i^	1.953 (4)	C30—C31	1.383 (6)
N9—C45	1.148 (5)	C30—H30	0.9300
N9—Cu3^iv^	2.004 (4)	C31—C32	1.475 (6)
N10—C46	1.154 (6)	C32—C33	1.390 (6)
N10—Cu3^iii^	1.979 (4)	C33—C34	1.358 (7)
C1—C2	1.355 (8)	C33—H33	0.9300
C1—H1	0.9300	C34—C35	1.374 (7)
C2—C3	1.360 (8)	C34—H34	0.9300
C2—H2	0.9300	C35—C36	1.365 (7)
C3—C4	1.386 (8)	C35—H35	0.9300
C3—H3	0.9300	C36—H36	0.9300
C4—C5	1.368 (7)	C37—C42	1.386 (6)
C4—H4	0.9300	C37—C38	1.389 (6)
C5—C6	1.478 (6)	C38—C39	1.377 (6)
C6—C7	1.382 (5)	C38—H38	0.9300
C7—C8	1.392 (6)	C39—C40	1.361 (7)
C7—H7	0.9300	C39—H39	0.9300
C8—C9	1.395 (6)	C40—C41	1.373 (8)
C8—C16	1.464 (6)	C40—H40	0.9300
C9—C10	1.382 (5)	C41—C42	1.385 (7)
C9—H9	0.9300	C41—H41	0.9300
C10—C11	1.483 (6)	C42—H42	0.9300
			
N5—Cu1—N2	175.67 (15)	C15—C14—C13	118.8 (5)
N5—Cu1—N1	105.11 (14)	C15—C14—H14	120.6
N2—Cu1—N1	77.42 (14)	C13—C14—H14	120.6
N5—Cu1—N4	77.58 (14)	N3—C15—C14	122.9 (4)
N2—Cu1—N4	105.65 (14)	N3—C15—H15	118.5
N1—Cu1—N4	99.01 (15)	C14—C15—H15	118.5
N5—Cu1—N3	100.08 (14)	C17—C16—C21	116.0 (4)
N2—Cu1—N3	77.53 (14)	C17—C16—C8	120.6 (4)
N1—Cu1—N3	154.77 (14)	C21—C16—C8	123.3 (4)
N4—Cu1—N3	84.97 (14)	C16—C17—C18	121.8 (5)
N5—Cu1—N6	77.55 (14)	C16—C17—H17	119.1
N2—Cu1—N6	99.24 (14)	C18—C17—H17	119.1
N1—Cu1—N6	86.46 (15)	C19—C18—C17	121.5 (5)
N4—Cu1—N6	155.11 (13)	C19—C18—H18	119.2
N3—Cu1—N6	100.42 (14)	C17—C18—H18	119.2
N8^i^—Cu2—N7^ii^	128.70 (19)	C20—C19—C18	117.2 (5)
N8^i^—Cu2—S2	104.74 (13)	C20—C19—H19	121.4
N7^ii^—Cu2—S2	108.40 (15)	C18—C19—H19	121.4
N8^i^—Cu2—S1	108.29 (15)	C19—C20—C21	120.8 (5)
N7^ii^—Cu2—S1	101.83 (14)	C19—C20—H20	119.6
S2—Cu2—S1	101.98 (7)	C21—C20—H20	119.6
N10^iii^—Cu3—N9^iv^	109.16 (16)	C16—C21—C20	122.4 (5)
N10^iii^—Cu3—S4	117.43 (13)	C16—C21—H21	118.8
N9^iv^—Cu3—S4	106.20 (12)	C20—C21—H21	118.8
N10^iii^—Cu3—S3	103.38 (12)	N4—C22—C23	122.5 (5)
N9^iv^—Cu3—S3	118.10 (12)	N4—C22—H22	118.8
S4—Cu3—S3	103.02 (5)	C23—C22—H22	118.8
C43—S1—Cu2	95.54 (17)	C22—C23—C24	119.6 (5)
C44—S2—Cu2	95.2 (2)	C22—C23—H23	120.2
C46—S3—Cu3	96.74 (15)	C24—C23—H23	120.2
C45—S4—Cu3	98.45 (16)	C25—C24—C23	119.3 (5)
C1—N1—C5	118.5 (4)	C25—C24—H24	120.4
C1—N1—Cu1	128.4 (4)	C23—C24—H24	120.4
C5—N1—Cu1	111.8 (3)	C24—C25—C26	118.8 (5)
C6—N2—C10	120.3 (4)	C24—C25—H25	120.6
C6—N2—Cu1	119.8 (3)	C26—C25—H25	120.6
C10—N2—Cu1	119.8 (3)	N4—C26—C25	121.7 (4)
C11—N3—C15	117.6 (4)	N4—C26—C27	114.6 (4)
C11—N3—Cu1	112.4 (3)	C25—C26—C27	123.7 (4)
C15—N3—Cu1	129.1 (3)	N5—C27—C28	120.6 (4)
C22—N4—C26	118.1 (4)	N5—C27—C26	115.1 (4)
C22—N4—Cu1	129.5 (3)	C28—C27—C26	124.2 (4)
C26—N4—Cu1	112.4 (3)	C27—C28—C29	120.3 (4)
C27—N5—C31	120.2 (3)	C27—C28—H28	119.8
C27—N5—Cu1	119.5 (3)	C29—C28—H28	119.8
C31—N5—Cu1	119.6 (3)	C30—C29—C28	118.0 (4)
C36—N6—C32	118.4 (4)	C30—C29—C37	121.3 (4)
C36—N6—Cu1	128.7 (3)	C28—C29—C37	120.7 (4)
C32—N6—Cu1	111.9 (3)	C31—C30—C29	120.1 (4)
C43—N7—Cu2^ii^	153.4 (4)	C31—C30—H30	120.0
C44—N8—Cu2^i^	156.0 (5)	C29—C30—H30	120.0
C45—N9—Cu3^iv^	154.1 (4)	N5—C31—C30	120.7 (4)
C46—N10—Cu3^iii^	158.2 (4)	N5—C31—C32	114.8 (4)
N1—C1—C2	123.1 (5)	C30—C31—C32	124.4 (4)
N1—C1—H1	118.5	N6—C32—C33	121.4 (4)
C2—C1—H1	118.5	N6—C32—C31	114.8 (4)
C1—C2—C3	119.0 (5)	C33—C32—C31	123.8 (4)
C1—C2—H2	120.5	C34—C33—C32	119.0 (5)
C3—C2—H2	120.5	C34—C33—H33	120.5
C2—C3—C4	119.1 (6)	C32—C33—H33	120.5
C2—C3—H3	120.5	C33—C34—C35	119.6 (5)
C4—C3—H3	120.5	C33—C34—H34	120.2
C5—C4—C3	119.1 (5)	C35—C34—H34	120.2
C5—C4—H4	120.5	C36—C35—C34	118.7 (5)
C3—C4—H4	120.5	C36—C35—H35	120.7
N1—C5—C4	121.3 (4)	C34—C35—H35	120.7
N1—C5—C6	114.7 (4)	N6—C36—C35	122.9 (5)
C4—C5—C6	124.1 (4)	N6—C36—H36	118.6
N2—C6—C7	120.9 (4)	C35—C36—H36	118.6
N2—C6—C5	114.9 (4)	C42—C37—C38	118.6 (4)
C7—C6—C5	124.3 (4)	C42—C37—C29	121.7 (4)
C6—C7—C8	120.7 (4)	C38—C37—C29	119.6 (4)
C6—C7—H7	119.6	C39—C38—C37	120.6 (4)
C8—C7—H7	119.6	C39—C38—H38	119.7
C7—C8—C9	117.0 (4)	C37—C38—H38	119.7
C7—C8—C16	121.6 (4)	C40—C39—C38	120.3 (5)
C9—C8—C16	121.3 (4)	C40—C39—H39	119.9
C10—C9—C8	120.2 (4)	C38—C39—H39	119.9
C10—C9—H9	119.9	C39—C40—C41	120.3 (5)
C8—C9—H9	119.9	C39—C40—H40	119.9
N2—C10—C9	120.9 (4)	C41—C40—H40	119.9
N2—C10—C11	115.0 (4)	C40—C41—C42	120.1 (5)
C9—C10—C11	124.1 (4)	C40—C41—H41	120.0
N3—C11—C12	122.1 (4)	C42—C41—H41	120.0
N3—C11—C10	114.5 (4)	C41—C42—C37	120.1 (5)
C12—C11—C10	123.4 (4)	C41—C42—H42	119.9
C11—C12—C13	119.1 (5)	C37—C42—H42	119.9
C11—C12—H12	120.5	N7—C43—S1	177.8 (5)
C13—C12—H12	120.5	N8—C44—S2	176.3 (7)
C14—C13—C12	119.5 (5)	N9—C45—S4	178.5 (5)
C14—C13—H13	120.3	N10—C46—S3	179.1 (4)
C12—C13—H13	120.3		
			
N8^i^—Cu2—S1—C43	159.5 (2)	C16—C8—C9—C10	179.5 (5)
N7^ii^—Cu2—S1—C43	21.6 (2)	C6—N2—C10—C9	1.2 (6)
S2—Cu2—S1—C43	−90.40 (18)	Cu1—N2—C10—C9	−175.3 (3)
N8^i^—Cu2—S2—C44	18.0 (3)	C6—N2—C10—C11	−180.0 (4)
N7^ii^—Cu2—S2—C44	158.2 (3)	Cu1—N2—C10—C11	3.6 (5)
S1—Cu2—S2—C44	−94.9 (3)	C8—C9—C10—N2	−0.5 (7)
N10^iii^—Cu3—S3—C46	9.68 (19)	C8—C9—C10—C11	−179.2 (4)
N9^iv^—Cu3—S3—C46	−110.9 (2)	C15—N3—C11—C12	0.3 (7)
S4—Cu3—S3—C46	132.43 (16)	Cu1—N3—C11—C12	−170.1 (4)
N10^iii^—Cu3—S4—C45	−110.1 (2)	C15—N3—C11—C10	179.6 (4)
N9^iv^—Cu3—S4—C45	12.3 (2)	Cu1—N3—C11—C10	9.3 (5)
S3—Cu3—S4—C45	137.04 (16)	N2—C10—C11—N3	−8.8 (6)
N5—Cu1—N1—C1	−1.3 (5)	C9—C10—C11—N3	170.0 (4)
N2—Cu1—N1—C1	−177.7 (5)	N2—C10—C11—C12	170.6 (5)
N4—Cu1—N1—C1	78.2 (5)	C9—C10—C11—C12	−10.7 (7)
N3—Cu1—N1—C1	175.5 (4)	N3—C11—C12—C13	−1.1 (8)
N6—Cu1—N1—C1	−77.4 (5)	C10—C11—C12—C13	179.6 (5)
N5—Cu1—N1—C5	165.4 (3)	C11—C12—C13—C14	0.7 (10)
N2—Cu1—N1—C5	−11.0 (3)	C12—C13—C14—C15	0.5 (10)
N4—Cu1—N1—C5	−115.1 (3)	C11—N3—C15—C14	1.0 (7)
N3—Cu1—N1—C5	−17.8 (6)	Cu1—N3—C15—C14	169.5 (4)
N6—Cu1—N1—C5	89.3 (3)	C13—C14—C15—N3	−1.4 (9)
N1—Cu1—N2—C6	7.5 (3)	C7—C8—C16—C17	1.2 (9)
N4—Cu1—N2—C6	103.5 (3)	C9—C8—C16—C17	−178.7 (6)
N3—Cu1—N2—C6	−175.4 (4)	C7—C8—C16—C21	178.8 (6)
N6—Cu1—N2—C6	−76.7 (3)	C9—C8—C16—C21	−1.1 (9)
N1—Cu1—N2—C10	−176.0 (3)	C21—C16—C17—C18	−0.4 (11)
N4—Cu1—N2—C10	−80.0 (3)	C8—C16—C17—C18	177.4 (6)
N3—Cu1—N2—C10	1.1 (3)	C16—C17—C18—C19	4.5 (12)
N6—Cu1—N2—C10	99.8 (3)	C17—C18—C19—C20	−5.0 (11)
N5—Cu1—N3—C11	177.8 (3)	C18—C19—C20—C21	1.6 (11)
N2—Cu1—N3—C11	−5.9 (3)	C17—C16—C21—C20	−3.0 (11)
N1—Cu1—N3—C11	0.9 (5)	C8—C16—C21—C20	179.3 (6)
N4—Cu1—N3—C11	101.4 (3)	C19—C20—C21—C16	2.4 (11)
N6—Cu1—N3—C11	−103.2 (3)	C26—N4—C22—C23	2.9 (8)
N5—Cu1—N3—C15	8.9 (4)	Cu1—N4—C22—C23	−179.6 (4)
N2—Cu1—N3—C15	−174.8 (4)	N4—C22—C23—C24	−1.1 (9)
N1—Cu1—N3—C15	−168.0 (4)	C22—C23—C24—C25	−1.3 (10)
N4—Cu1—N3—C15	−67.6 (4)	C23—C24—C25—C26	1.8 (9)
N6—Cu1—N3—C15	87.9 (4)	C22—N4—C26—C25	−2.3 (7)
N5—Cu1—N4—C22	179.2 (5)	Cu1—N4—C26—C25	179.8 (4)
N2—Cu1—N4—C22	−3.8 (5)	C22—N4—C26—C27	176.8 (4)
N1—Cu1—N4—C22	75.6 (4)	Cu1—N4—C26—C27	−1.1 (5)
N3—Cu1—N4—C22	−79.3 (4)	C24—C25—C26—N4	0.1 (8)
N6—Cu1—N4—C22	176.7 (4)	C24—C25—C26—C27	−179.0 (5)
N5—Cu1—N4—C26	−3.2 (3)	C31—N5—C27—C28	−3.3 (6)
N2—Cu1—N4—C26	173.8 (3)	Cu1—N5—C27—C28	166.9 (3)
N1—Cu1—N4—C26	−106.8 (3)	C31—N5—C27—C26	179.3 (4)
N3—Cu1—N4—C26	98.3 (3)	Cu1—N5—C27—C26	−10.5 (5)
N6—Cu1—N4—C26	−5.7 (6)	N4—C26—C27—N5	7.1 (6)
N1—Cu1—N5—C27	103.7 (3)	C25—C26—C27—N5	−173.7 (5)
N4—Cu1—N5—C27	7.6 (3)	N4—C26—C27—C28	−170.2 (4)
N3—Cu1—N5—C27	−75.0 (3)	C25—C26—C27—C28	8.9 (7)
N6—Cu1—N5—C27	−173.5 (3)	N5—C27—C28—C29	0.8 (7)
N1—Cu1—N5—C31	−86.1 (3)	C26—C27—C28—C29	178.0 (4)
N4—Cu1—N5—C31	177.8 (3)	C27—C28—C29—C30	2.1 (7)
N3—Cu1—N5—C31	95.3 (3)	C27—C28—C29—C37	−176.1 (4)
N6—Cu1—N5—C31	−3.2 (3)	C28—C29—C30—C31	−2.6 (6)
N5—Cu1—N6—C36	−172.3 (5)	C37—C29—C30—C31	175.6 (4)
N2—Cu1—N6—C36	10.6 (5)	C27—N5—C31—C30	2.8 (6)
N1—Cu1—N6—C36	−66.0 (5)	Cu1—N5—C31—C30	−167.4 (3)
N4—Cu1—N6—C36	−169.8 (4)	C27—N5—C31—C32	179.9 (4)
N3—Cu1—N6—C36	89.5 (5)	Cu1—N5—C31—C32	9.7 (5)
N5—Cu1—N6—C32	−4.2 (3)	C29—C30—C31—N5	0.2 (6)
N2—Cu1—N6—C32	178.8 (3)	C29—C30—C31—C32	−176.6 (4)
N1—Cu1—N6—C32	102.2 (3)	C36—N6—C32—C33	0.4 (7)
N4—Cu1—N6—C32	−1.7 (6)	Cu1—N6—C32—C33	−169.1 (4)
N3—Cu1—N6—C32	−102.3 (3)	C36—N6—C32—C31	179.6 (4)
C5—N1—C1—C2	0.7 (9)	Cu1—N6—C32—C31	10.1 (5)
Cu1—N1—C1—C2	166.6 (5)	N5—C31—C32—N6	−13.1 (6)
N1—C1—C2—C3	−0.6 (11)	C30—C31—C32—N6	163.9 (4)
C1—C2—C3—C4	−0.5 (11)	N5—C31—C32—C33	166.1 (4)
C2—C3—C4—C5	1.4 (11)	C30—C31—C32—C33	−17.0 (7)
C1—N1—C5—C4	0.3 (8)	N6—C32—C33—C34	−1.1 (8)
Cu1—N1—C5—C4	−167.9 (5)	C31—C32—C33—C34	179.8 (5)
C1—N1—C5—C6	−179.2 (4)	C32—C33—C34—C35	1.1 (9)
Cu1—N1—C5—C6	12.6 (5)	C33—C34—C35—C36	−0.6 (9)
C3—C4—C5—N1	−1.3 (9)	C32—N6—C36—C35	0.2 (8)
C3—C4—C5—C6	178.2 (6)	Cu1—N6—C36—C35	167.7 (5)
C10—N2—C6—C7	−0.9 (6)	C34—C35—C36—N6	−0.1 (10)
Cu1—N2—C6—C7	175.5 (3)	C30—C29—C37—C42	36.2 (7)
C10—N2—C6—C5	−179.6 (4)	C28—C29—C37—C42	−145.7 (5)
Cu1—N2—C6—C5	−3.1 (5)	C30—C29—C37—C38	−144.1 (4)
N1—C5—C6—N2	−7.1 (6)	C28—C29—C37—C38	34.0 (6)
C4—C5—C6—N2	173.4 (5)	C42—C37—C38—C39	−0.2 (7)
N1—C5—C6—C7	174.3 (4)	C29—C37—C38—C39	−179.9 (4)
C4—C5—C6—C7	−5.2 (8)	C37—C38—C39—C40	−0.6 (7)
N2—C6—C7—C8	0.0 (7)	C38—C39—C40—C41	0.9 (8)
C5—C6—C7—C8	178.5 (4)	C39—C40—C41—C42	−0.5 (9)
C6—C7—C8—C9	0.7 (6)	C40—C41—C42—C37	−0.2 (9)
C6—C7—C8—C16	−179.2 (5)	C38—C37—C42—C41	0.6 (8)
C7—C8—C9—C10	−0.4 (6)	C29—C37—C42—C41	−179.8 (5)

Symmetry codes: (i) −*x*, −*y*+2, −*z*+2; (ii) −*x*, −*y*+1, −*z*+2; (iii) −*x*, −*y*, −*z*+1; (iv) −*x*, −*y*+1, −*z*+1.

### Hydrogen-bond geometry (Å, °)

**Table:**

*D*—H···*A*	*D*—H	H···*A*	*D*···*A*	*D*—H···*A*
C28—H28···S3^v^	0.93	2.82	3.697 (5)	158
C36—H36···S1	0.93	2.81	3.657 (5)	152

Symmetry codes: (v) −*x*+1, −*y*+1, −*z*+1.
